# Dressed in Collagen: 2D and 3D Cardiac Fibrosis Models

**DOI:** 10.3390/ijms26073038

**Published:** 2025-03-26

**Authors:** Maria Cardona-Timoner, Rita N. Gomes, Diana S. Nascimento

**Affiliations:** 1 Institute for Research and Innovation in Health (i3S), University of Porto, 4200-135 Porto, Portugal; mtimoner@i3s.up.pt (M.C.-T.); argomes@i3s.up.pt (R.N.G.); 2 Instituto Nacional de Engenharia Biomédica (INEB), University of Porto, 4200-135 Porto, Portugal; 3 Instituto de Ciências Biomédicas Abel Salazar (ICBAS), University of Porto, 4050-313 Porto, Portugal; 4 Faculdade de Engenharia da Universidade do Porto (FEUP), 4200-465 Porto, Portugal

**Keywords:** cardiac fibrosis, cardiac fibroblasts, in vitro fibrosis models, disease modeling

## Abstract

Cardiovascular diseases (CVD), the leading cause of death worldwide, and their strong association with fibrosis highlight the pressing need for innovative antifibrotic therapies. In vitro models have emerged as valuable tools for replicating cardiac fibrosis ‘in a dish’, facilitating the study of disease mechanisms and serving as platforms for drug testing and development. These in vitro systems encompass 2D and 3D models, each with its own limitations and advantages. 2D models offer high reproducibility, cost-effectiveness, and high-throughput capabilities, but they oversimplify the complex fibrotic environment. On the other hand, 3D models provide greater biological relevance but are more complex, harder to reproduce, and less suited for high-throughput screening. The choice of model depends on the specific research question and the stage of drug development. Despite significant progress, challenges remain, including the integration of immune cells in cardiac fibrosis and optimizing the scalability and throughput of highly biomimetic systems. Herein, we review recent in vitro cardiac fibrosis models, with a focus on their shared characteristics and remaining challenges, and explore how in vitro fibrosis models of other organs could inspire novel approaches in cardiac research, showcasing potential strategies that could be adapted to refine myocardial fibrosis models.

## 1. Introduction

The association between fibrosis and disease dates back to the 19th century when Rudolf Virchow described it in his influential book, *Die Cellularpathologie* (1858, Berlin). Fibrosis is an excessive deposition of extracellular matrix (ECM) proteins and an important cause of organ disfunction across various organs [1]. In the heart, myocardial fibrosis represents an expansion of the cardiac interstitium due to the accumulation of ECM proteins. These ECM changes affect the heart both macroscopically, leading to increased stiffness and hypertrophy, and at the molecular level, by disrupting anisotropy and electrical coupling, ultimately impairing cardiac function [2]. As such, cardiac fibrosis is a strong predictor of poor clinical outcomes in most CVD [3].

There are three main contributors to cardiac fibrosis: cellular components, paracrine signals, and the ECM. Among the four major cardiac cell types, i.e., cardiomyocytes (CM), cardiac fibroblasts (CF), endothelial cells, and immune cells, CF are the primary mediators of fibrosis, influencing both cardiac function and CM behavior. When exposed to profibrotic stimuli (such as mechanical or biochemical signals), CF proliferate, become activated, differentiate into myofibroblasts, and secrete excessive ECM. This aberrant ECM deposition increases tissue stiffness, reduces compliance, and disrupts myocardial architecture by forming collagen bundles that separate CM strands. Such structural disorganization slows and fragments electrical conduction, generating discontinuous and through re-entry circuits that promote arrhythmogenesis [4,5]. At the same time, CF become more abundant and aberrantly couple with CM via gap junctions and ion channels, further disturbing electrical signaling [6]. Additionally, CM rely on the ECM for proper sarcomere organization and mechanical force transmission. Disruptions in ECM composition impair this process, leading to dysfunction in CM mechanics and compromised contractile force propagation [7,8,9,10].

Cardiac fibrosis manifests in two main forms: reparative and interstitial. Reparative fibrosis is the body’s failed attempt to protect the heart from acute damage. Upon myocardial infarction (MI), CM death triggers myofibroblast activation [11], leading to the production of collagen-based scar tissue, which temporarily maintains structural integrity and prevents cardiac rupture [12]. However, this non-contractile scar tissue eventually leads to impaired cardiac function, serving as only a temporary solution [13]. In contrast, chronic conditions like left ventricular pressure overload result in interstitial and perivascular fibrosis, which stiffens the heart tissue and reduces compliance without CM loss [14]. Thus, this fibrotic ECM, produced by CF and affecting cardiac and CM function, can be conceptualized as a chronic, cyclical process of repair that degenerates into a pathological state. Ongoing stress and signaling activate CF, leading to excessive ECM deposition, which stiffens tissue and further amplifies and maintains a vicious cycle of CF activation, perpetuating dysfunction and damage to the heart [15].

CVD remain the leading cause of death worldwide and are still on the rise, especially in developing countries [16]. The aging global population has also contributed to an increase in both acute and chronic heart failure [17]. Consequently, CVD urgently require new treatments including those able to control and/or inhibit cardiac fibrosis. While some antifibrotic treatments have been approved for pulmonary diseases [18], no specific therapies are currently available for cardiac fibrosis. Developing effective therapies begins with in vitro testing, as these models offer controlled environments to investigate disease mechanisms, screen potential drugs, and accelerate the development of therapies for cardiac fibrosis.

**Figure 1:**
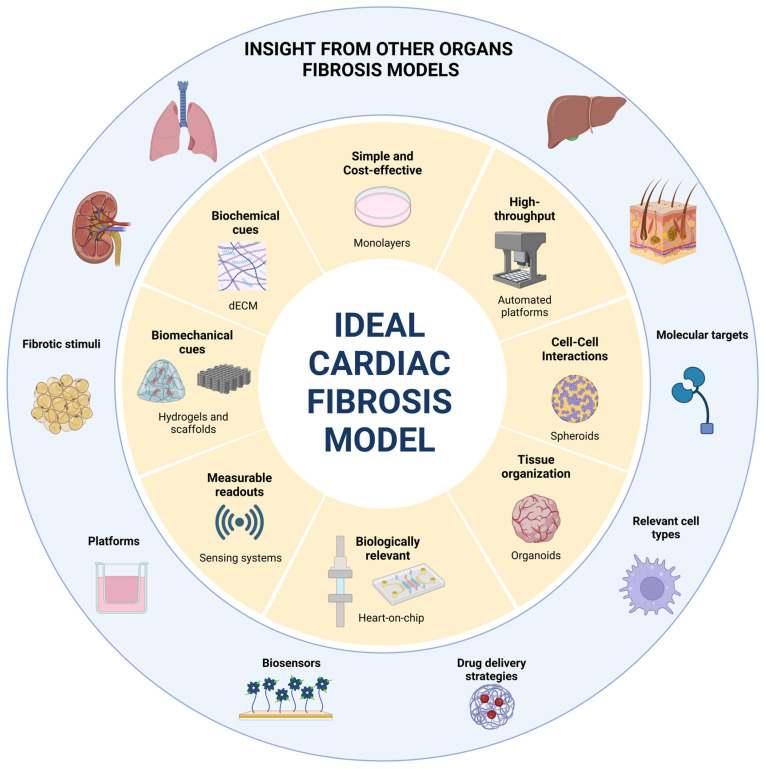
**Features of cardiac fibrosis models and insights from other organs.** The inner circle represents key features that cardiac fibrosis models include, encompassing simplicity and cost-effectiveness, biochemical and biomechanical cues, measurable readouts, biological relevance, tissue organization, enhanced cell–cell interactions, and high-throughput capabilities. Below each attribute, the most suitable model type is indicated. The outer circle highlights fibrosis models from other organs, emphasizing features that could be learned from them and incorporated into cardiovascular research to further refine in vitro cardiac fibrosis models. Created in BioRender. Cardona, M. (2025) https://BioRender.com/r40s644 (accessed on 22 March 2025).

This work reviews current in vitro models of cardiac fibrosis, focusing on both two-dimensional (2D) and three-dimensional (3D) systems, identifying their advantages, limitations, and applications in order to guide researchers in selecting the most suitable cardiac fibrosis models for their specific scientific objectives. Additionally, it explores in vitro fibrosis models developed for other organs, identifying methodologies, creative ideas, and alternative approaches that could inspire advances in cardiac research (Figure 1).

## 2. Molecular and Cellular Players in Cardiac Fibrosis

### 2.1. Myofibroblasts

**Figure 2:**
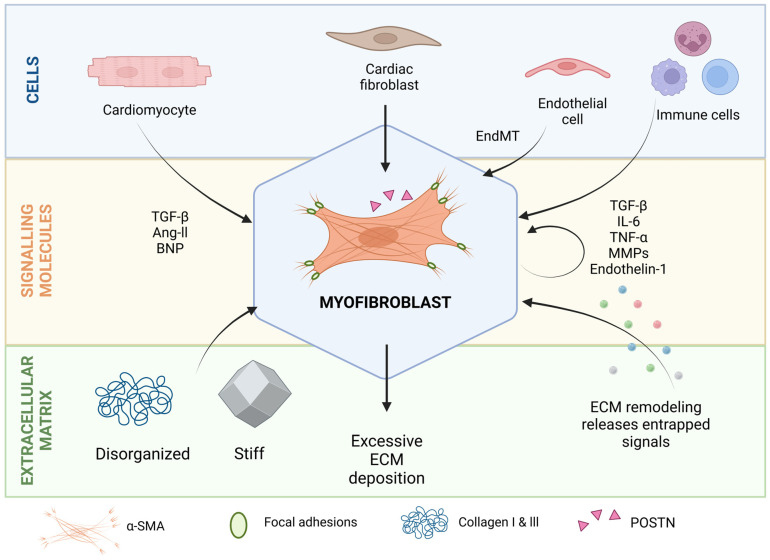
**Cardiac myofibroblast activation and regulation in fibrosis.** Myofibroblasts originate from various cellular sources, primarily cardiac fibroblasts, but also from other cell types such as endothelial cells undergoing endothelial-to-myofibroblast transition (EndMT). Following cardiac injury, additional cells within the myocardial parenchyma, including cardiomyocytes (CM) and immune cells, contribute to myofibroblast activation through signaling molecules such as transforming growth factor-beta (TGF-β), angiotensin II (Ang-II), and brain natriuretic peptide (BNP), as well as pro-inflammatory cytokines, including interleukin 6 (IL-6), tumor necrosis factor-alpha (TNF-α), matrix metalloproteinases (MMPs), and endothelin-1. Notably, myofibroblasts also secrete some of these signaling molecules, reinforcing a positive feedback loop that sustains their activation. Once activated, myofibroblasts play a central role in cardiac fibrosis by producing and depositing excessive extracellular matrix (ECM) proteins, leading to increased matrix stiffness and structural disorganization. Additionally, ECM remodeling releases sequestered signaling molecules, further amplifying fibrotic signaling and maintaining myofibroblast activation. The complex interplay between cellular components, signaling pathways, and ECM alterations highlights key mechanisms driving cardiac fibrosis and represents potential therapeutic targets for antifibrotic interventions. Created in BioRender. Cardona, M. (2025) n21f997.

When the heart is subjected to stress conditions, such as MI or pressure overload, myocardial resident CF are activated by damage-associated molecular patterns (DAMPs), inflammatory signals, and biomechanical cues that drive their recruitment, proliferation, and differentiation into myofibroblasts [19,20] (Figure 2). Myofibroblasts are distinct from quiescent tissue-resident CF by exhibiting a contractile phenotype, marked by the expression of α-smooth muscle actin (α-SMA) and periostin (POSTN) [21], and show an enhanced capacity to synthesize ECM proteins, especially collagen type I and III [22,23]. Beyond these molecular changes, myofibroblasts demonstrate altered biological behaviors, including increased proliferation, migration, or secretion of cytokines like transforming growth factor beta (TGF-β) and interleukin 6 (IL-6) [24]. Importantly, CF activation is not a one-step phenotypic switch, but rather a dynamic process with intermediate states like proto-myofibroblasts, which are activated CF but lack α-SMA expression [25]. Furthermore, myofibroblast activation is a transient phenotype that can be reversed under certain conditions [26]. Whether reverted fibroblasts are similar to quiescent fibroblasts is still unknown [27]. However, in a chronic and stable activation state, myofibroblasts can transition to a matrifibrocyte stage, where the expression of genes typical of bone and cartilaginous tissues, such as *Cilp2* and *Comp*, is increased [28]. In this state, activation is much less likely to be reverted [27].

### 2.2. Paracrine Signals

Although myofibroblasts are central to cardiac fibrosis, they do not act in isolation. CM, EC, and immune cells secrete a variety of molecules that contribute to fibroblast activation and fibrotic progression (Figure 2). After an acute injury, necrotic CM release DAMPs, which trigger an inflammatory response [29]. The response to these signals by the immune system includes the release of cytokines and inflammatory mediators. The most relevant soluble signals driving CF activation include growth factors like TGF-β and platelet-derived growth factors (PDGFs), cytokines such as interleukin 1β and 6 (IL-6) and tumor necrosis factor-α (TNF-α), and hormones like aldosterone and angiotensin II (Ang II) [30].

In a healthy heart, TGF-β is secreted by macrophages, fibroblasts, platelets, vascular cells, and CM in a latent complex that remains entrapped in the cardiac ECM [31]. Upon injury, the latent complex is cleaved by proteases, releasing its active form and stimulating CF activation and myofibroblast differentiation [32].

Ang II, a hormone of the renin–angiotensin–aldosterone system, can also be released by CM upon cardiac injury. Ang II has a direct effect on CM and CF by inducing the expression of TGF-β and stimulating CF (or myofibroblasts) proliferation, migration, and ECM production, with the TGF-β signaling pathway as a key intermediary [30]. Similarly, aldosterone, another hormone regulating fluid homeostasis, directly promotes profibrotic effects. It induces the expression of TGF-β, connective tissue growth factor (CTGF), collagen type I, fibronectin, and endothelin-1. Additionally, it indirectly modulates Ang II levels and receptor activity, further amplifying its fibrotic influence [33,34].

Proinflammatory cytokines, primarily secreted by immune cells, further exacerbate fibrosis through indirect activation of mediators such as TGF-β. Additionally, IL-6 and TNF-α directly contribute to fibrosis by promoting the proliferation of resident CF and modulating matrix metalloproteinases (MMPs) expression, which can subsequently activate TGF-β and remodel the ECM [19,35].

Altogether, the TGF-β pathway is central to all these different soluble stimuli driving CF activation, functioning as the primary signaling hub within the broader fibrotic machinery and emphasizing the TGF-β pathway as a key therapeutic target [32]. However, growing evidence highlights the intricate crosstalk between multiple pathways, including TGF-β, Ang II, and Wnt signaling, which together form a highly inter-connected regulatory network that governs fibrosis progression [36,37,38].

### 2.3. ECM as a Source of Biomechanical Cues and Signaling Molecules That Regulate Cell Profibrotic Activity

In addition to the released soluble signals, it is important to consider that the ECM can also act as a reservoir of signaling molecules, as is the case of latent TGF-β [39] (Figure 2). Furthermore, during ECM remodeling and degradation, peptides are released by partial proteolysis of ECM macromolecules, commonly termed ‘matrikines’ [40,41]. Matrikines are bioactive signals that influence the behavior of surrounding cells. For example, endotrophin, a type VI collagen-derived fragment, is increased in patients with heart failure with preserved ejection fraction or other fibro-inflammatory diseases. Interestingly, it has been demonstrated that endotrophin can activate type I collagen synthesis in CF, pinpointing its possible role as a causal biomarker, both reflecting and contributing to disease progression [40].

As mentioned, fibrosis is characterized by excessive deposition of ECM, mainly fibrillar collagens and fibronectin, leading to a loss of tissue anisotropy (the property of having different structural or functional characteristics depending on the direction, which is important for proper tissue organization and function) and increased stiffness (Figure 2). CF oversee maintaining ECM homeostasis, meaning that they are responsible for producing this ECM, but also for sensing it. Mechanotransduction is the process by which cells detect and react to mechanical forces, converting physical signals into biochemical responses. These appear to be the main mechanisms behind interstitial fibrosis [42]. Several pathways have been proposed in the process of cardiac fibrosis mediated by mechanotransduction [43]. These include the regulation of the Hippo pathway (controls organ size and cell proliferation, while also responding to mechanical stress at the cell–cell adhesion level), the RhoA/ROCK pathway (involved in regulating cell shape, movement, and contraction in response to mechanical stress), and the Wnt/β-catenin pathway (regulates cell growth, development, and tissue repair, influenced by mechanical forces) [44,45,46,47]. For example, after MI, myocardial compliance differs significantly between the scar tissue area (∼10 kPa) and the surrounding intact area (∼40 kPa) [48]. Various studies have pinpointed that fibroblast differentiation towards myofibroblast can be triggered by stiff matrices [48,49,50]. As such, CF are responsible for both sensing and synthesizing the fibrotic ECM in a feed-forward loop that sustains the fibrotic process [15]. Yet, the question that remains is: how can this vicious cycle be resolved?

## 3. Main Characteristics of an Ideal In Vitro Model of Cardiac Fibrosis

**Figure 3:**
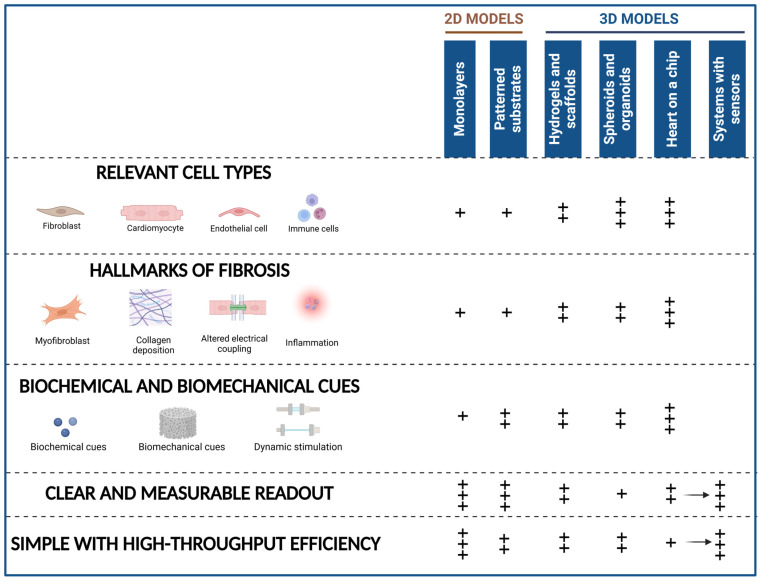
**Advantages and limitations of 2D and 3D models in fibrosis studies.** This figure provides a comparative overview of 2D and 3D in vitro models of cardiac fibrosis, highlighting their ability to mimic the features of an ideal fibrosis model. The “+” symbols denote the extent of representation, with “+” indicating minimal resemblance and “+++” indicating a higher degree of biomimicry. The key aspects of an ideal fibrosis model represented include: Relevant cell types (cardiac fibroblasts (CF), cardiomyocytes (CM), endothelial cells, and immune cells), Hallmarks of Fibrosis (myofibroblast activation, collagen deposition, altered electrical coupling, and inflammation), Biochemical and Biomechanical Cues, Clear and Measurable Readouts, and High-throughput Efficiency. In the image, figures/drawings within each of the features of an ideal model positioned further to the right indicate greater biomimicry and often involve the incorporation of the foundational components shown to their left. Created in BioRender. Cardona, M. (2025) https://BioRender.com/v55r799 (accessed on 22 March 2025).

An ideal model of cardiac fibrosis would recreate the complex fibrotic environment in vitro, including fibroblast activation and myofibroblast differentiation, excessive ECM deposition with altered mechanical properties, abnormal electrical coupling and contractility, and inflammation. In addition to replicating the pathological fibrosis response in a dish, this model should ensure reproducibility and allow for a quantifiable assessment of the response. Although such a model has yet to be developed, important criteria can be defined, some of which are more easily attainable through the design of 2D models, whereas others are only possible with more complex 3D systems (Figure 3):

(1) Include cellular populations involved in cardiac fibrosis, namely CF (including myofibroblasts), CM, EC, and immune cells.
(2) Accurately model the dynamic ECM remodeling with excessive collagen deposition, increased stiffness, and the release of bioactive factors from the matrix. Additionally, it should emulate the mechanical and electrical properties of fibrotic tissue.
(3) Consider the variety of triggers implicated in cardiac fibrosis. This should encompass biochemical and biomechanical cues but also the hypoxia or reperfusion conditions that naturally occur during MI.
(4) Have clear, measurable, and objective readouts of fibroblast activation, ECM deposition, electrical activity, and contractile behavior or an objective measure of a certain behavior (by including sensing systems).
(5) Be reproducible, cost-effective, and have high-throughput capabilities.

## 4. 2D and 3D In Vitro Models of Cardiac Fibrosis

Drug efficacy and toxicity during the preclinical stages rely heavily on the combination of in vivo animal studies and in vitro cell culture analyses. While animal models have historically been considered the gold standard for preclinical testing, they pose several challenges, including ethical concerns, high costs, long experimental timelines, and limited applicability to human physiology. As an alternative, in vitro cellular models are increasingly utilized, offering a range of formats that cater to different research needs [51].

In 2D models, cells are cultured on tissue culture polystyrene (TCP) plates, forming a monolayer. Their main advantage is their low cost, high-throughput possibilities, and simple analysis. However, they poorly replicate the ECM and cellular interactions of the native tissue, lacking structural complexity and physiological relevance. Alike traditional 2D cell cultures, 3D models attempt to provide a more realistic representation of the in vivo environment, by replicating topographical, electromechanical, and biochemical cues of the native tissue, increasing translatability to real-life conditions [52]. This can be achieved by embedding cells in or on a 3D matrix that mimics the healthy or diseased ECM. These systems often combine mechanical and biochemical cues, mimicking the dynamic processes in living tissues. However, given their complexity, 3D models are commonly expensive and labor-intensive, limiting high-content analysis [53]. In this review, models cultured on patterned substrates, microfluidic systems, or chips are also classified as 3D models. While some of these lack full three-dimensionality, they incorporate complex and engineered environments that more closely resemble native tissue structures and functions compared to monolayers.

**Figure 4:**
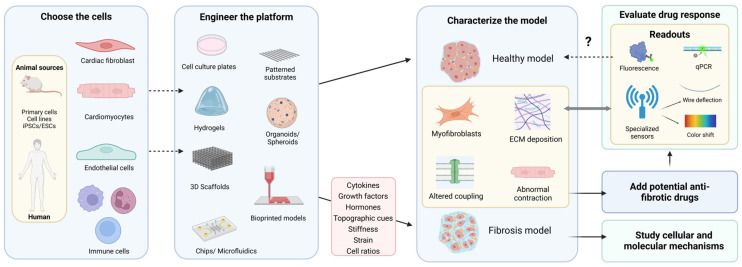
**Engineering cardiac fibrosis models**. This figure illustrates the pipeline to create an in vitro model for cardiac fibrosis. It begins with the selection of cells, which include cardiac fibroblasts, cardiomyocytes, endothelial cells, and immune cells. The cells can be obtained from primary sources, established cell lines, or induced pluripotent stem cells (iPSCs)/embryonic stem cells (ESCs). These cells are then incorporated into the chosen platform, which can range from simple cell culture plates and patterned substrates to more advanced 3D scaffolds, hydrogels, organoids/spheroids, bioprinted models, or microfluidic chips. The fibrotic model is then subjected to one or more profibrotic stimuli, and both the healthy and fibrotic models are characterized by assessing features such as myofibroblast activation, ECM deposition, altered coupling, and abnormal contraction. At this stage, the model can be used to investigate fundamental aspects of the pathology or serve as a platform to assess the antifibrotic potential of therapeutic candidates. In the latter case, some characterization parameters are re-evaluated (for example, by fluorescence imaging, quantitative PCR (qPCR), wire deflection measurements, specialized biosensors, or colorimetric shifts), providing a readout for assessing the effects of the antifibrotic treatment. The dashed arrow between the healthy model and drug evaluation highlights a key goal—assessing whether antifibrotic treatments can prevent or reverse fibrosis, making the fibrotic model resemble the healthy one. Created in BioRender. Cardona, M. (2025) https://BioRender.com/s85b878 (accessed on 22 March 2025).

2D or 3D models are equally valuable. It is the scientific question of what should determine the best platform to address it. Once decided, both approaches follow a shared pipeline to develop a robust and well-characterized in vitro model that effectively mimics the fibrotic environment. This process starts with cell and platform selection, followed by stimulation to induce fibrotic or healthy phenotypes, characterization of the resulting models, and finally, drug testing or basic biology studies (Figure 4). The following sections provide an overview of the existing methodologies used for each of these steps within the context of 2D and 3D models of cardiac fibrosis.

### 4.1. Cellular Components

The most used cell types are CF, followed by their combination with CM and, in some cases, EC. These cells can be either derived from human sources, which add translatability to the model, such as human induced pluripotent stem cell (hiPSC)-derived cardiac cells [54] or commercially available human primary CF (hCF) [55], or from animal sources, commonly primary CF isolated from murine hearts [56]. Importantly, since 3D models aim to be a step closer to clinical translation, most of them use cells from human sources rather than from animal ones (Table 1). However, it is essential to acknowledge certain limitations and variability associated with these cellular sources. For example, primary CF exhibit significant variability introduced by factors like age, isolation, culture conditions [57], and external stimuli [58,59]. Therefore, careful characterization of the basal fibroblast activation state is necessary, as factors like passage number or culture in stiff matrices have been reported to induce myofibroblast differentiation [59,60]. Additionally, their purity should be confirmed through the use of CF-specific markers such as discoidin domain receptor 2 (DDR2) [61], CD90 [62], and PDGFRα [63]. On a side note, while patient-derived hCF would be a valuable tool for replicating the real stimuli that CF experience during fibrotic events, their use remains uncommon due to the challenges in obtaining them, including limited donor availability and the invasive nature of sample collection. However, some studies have successfully utilized hCF derived from hiPSCs obtained from patients with specific genetic conditions [64].

While CF monocultures can replicate certain fibrotic processes [65], co-culturing CF with CM or EC adds biological relevance. Adult CM are considered terminally differentiated cells; thus, they cannot be expanded at appreciable rates from cardiac biopsies. As a result, hiPSCs or human embryonic stem cells are often used for differentiation into CM, although they have also been directly reprogrammed from CF (induced cardiomyocytes), which could closely resemble adult CM when compared to iPSC-CM [66]. Importantly, reprogrammed or differentiated cells always require marker validation (e.g., cardiac troponin T (cTnT)) to confirm their identity. As a co-culture example, H. Iseoka et al. [67] proposed a multicellular 2D fibrosis model consisting of hiPSC-CM and non-CM (positive for fibroblastic or endothelial markers). By combining different cellular types, this fibrosis model evaluated how profibrotic stimuli impact not only the expression of fibrosis-associated genes but also hiPSC-CM contractile behavior. Although there are not many 2D co-culture models specifically designed to study cardiac fibrosis, there are various examples used to study other cardiac processes [68]. Co-culture systems offer valuable insights into cell–cell interactions; however, the majority of these models are in 3D.

**Table 1:** **2D and 3D models of cardiac fibrosis.** References are not provided for commonly used stimuli, characterization techniques, or readouts. Representative articles are provided in the “Reference Example” column. Distinct or less frequently used stimuli, characterization approaches, and readouts are individually referenced.

Model		Cells	Stimuli	Characterization	Readout	Tested Molecules	Example References
**Monolayers**		CF (Human) [55,69] (Rat) [56,70]	TFG-β Ang ll [56,71]	**Proliferation and Viability:** Viability **Cell Markers:** α-SMA, POSTN, phalloidin [59] **ECM Remodeling:** Fibronectin, collagen type I and type III, collagen maturity (resistance to digestion, cell surface biotinylation experiments), deposition of extracellular collagen (immunofluorescence for glycosaminoglycans) [55] **Inflammatory markers:** Inflammatory protein expression panel, intracellular ROS [69] **Secreted Molecules:** TGF-β, BNP secretion **Phenotypic changes throughout culture:** [59]	**Proliferation, viability, and migration:** Cell viability, cytotoxicity, proliferation and migration [54,70,71,72] **Cell Markers:** α-SMA **ECM Remodeling:** Collagen type I, telo-collagen IαI expression and secretion [65], MMP-2 and MMP-9 activity (gelatin zymography) [72] **Inflammatory markers:** Inflammatory protein expression panel, intracellular ROS **Activated Pathways:** Wnt1, β-catenin **Others:** Proline incorporation [70]	**Small molecules** Pirfenidone [54] SB431542, 21 small molecules and 2 proteins previously reported to confer antifibrotic effects in vivo, compound ESI09 [55] Molecules based on Tranilast’s core structure (FT011) [56] Src inhibitor WH-4 023 [73] **Natural compounds** Ellagic acid, punicic acid [69] Curcumin [72] Quercetin dihydrate [71] Molecular similarities of bufalin and lycorine [74] **Libraries of compounds** Libraries of 5000 preclinical and clinical compounds [54] Library of natural compounds [75]	[54] [55] [56] [59] [71] [70] [72] [69] [75] [73] [74]
		CF, CM and EC (Human)	TFG-β	**Proliferation and Viability:** Viability **Cell Markers:** Vimentin, CD90, CD31, cTnT, TGF-β receptor (TGF-βR), antifibrotic factor HGF receptor (c-met) **ECM Remodeling:** Collagen type I, collagen type III, fibronectin, MMP-2, PCR array of ECM-related genes **Secreted molecules:** TFG-β expression	**ECM Remodeling:** Collagen type I, Collagen type III, Fibronectin, MMP-2, PCR array of ECM related genes **Secreted molecules:** TFG-β expression **Contraction and relaxation behavior**	HGF ONO-1301 Camostat mesilate Pirfenidone	[67]
**Hydrogels/Scaffolds**	Patterned substrates (PEG hydrogel matrix)	CF (Rat)	Stiffness	**Purity of Rat CF:** DDR2 expression **Cell Markers:** α-SMA, F-actin **ECM Remodeling:** Collagen, fibronectin **Proliferation and Viability:** Cell proliferation, cell migration	**Cell Markers:** α-SMA, F-actin **ECM Remodeling:** Collagen, fibronectin	ROCK inhibitor (Y27632)	[60]
	Structural color hydrogels	CM and CF (Human)	CM:CF ratio (Healthy 3:1-Fibrotic 1:3)	**Proliferation and Viability:** Cell proliferation, cell viability **Cell Markers:** α-SMA **ECM Remodeling:** Collagen type I **Mechanical Properties:** Microtissue stiffness, passive tension **Secreted Molecules:** BNP secretion **Hydrogel color shift properties**	**Functional/Contractile Properties** (Contraction = Color shift)	Losartan Saracatinib Relaxin-2 Pirfenidone Nintedanib	[76]
	Hydrogels (GelMa)	CF (Human)	TFG-β	**Proliferation and Viability:** Cell spreading in hydrogel, viability **Cell Markers:** α-SMA, vimentin, fibroblast-specific protein (FSP) **ECM Remodeling:** Collagen IαI/IIIαI **Mechanical Properties:** Mechanical properties of hydrogels (elastic modulus, mass swelling ratio, and pore size)	**Cell Markers:** α-SMA **ECM Deposition:** Collagen IαI/IIIαI	Paracrine effects of cardiac progenitor cells	[26]
		CF and CM (Rat) [77] (Human) [78]	Stiffness TGF-β [78]	**Proliferation and Viability:** Cell spreading in hydrogel, viability, proliferation, metabolic activity **Cell Markers:** α-SMA, vimentin **ECM Remodeling:** Collagen type I and type III **Hydrogel Characterization:** Mechanical properties (elastic modulus, stiffness), mass swelling ratio, pore size, morphology (SEM) **Contraction and Electrophysiological Behavior:** Beating pattern (beats per minute, synchronicity), maturation phase (time for cells to become fully functional after fabrication), hydrogel contraction test [54,77] **Calcium Handling/Dynamics:** Cell coupling (electromechanical coupling, calcium transients) **Other:** Targeted proteomics	**Cell Markers:** α-SMA **ECM Deposition:** Fibronectin, collagen type I and type III, MMP-2, POSTN **Contraction and Electrophysiological Behavior:** Beating pattern **Other:** Targeted proteomics	Isoprenaline (to assess physiological beating response) [77] Pirfenidone	[54] [77] [78]
	Scaffolds	CF (Human)	Random nanofibrous organization and stiffness	**Proliferation and Viability:** Cytocompatibility, cytotoxicity **Scaffold Characterization:** Nanofiber orientation, porosity, area (SEM), mechanical properties (Young modulus) Surface coating and grafting efficiency **Cell Markers:** Cell distribution on scaffold, α-SMA (fibroblast activation on scaffold)	**Cell Markers:** α-SMA **ECM Deposition:** Fibronectin, collagen type I, collagen type III	Tranilast [79]	[79] [80]
**Spheroids**	Three-cell-type spheroids	CF, CM and EC (Rat)	Phenylephrine-induced cardiac hypertrophy [81] TGF-β + Doxycycline [82]	**Proliferation and Viability:** Cell death (Calcein/PI), mitochondrial and membrane potential, calcium concentration, apoptosis, and remodeling of the spheroid **Structural/Phenotypic Markers:** Markers of CMs, CFs, and ECs in the microspheres, HE staining (muscle fibers), expression of CTGF, fibronectin, TGFβ **Dimensional Properties:** Diameter **Functional Properties:** Beating frequency **ECM and Histological Analysis:** ECM deposition (histological analysis using PicroSirius Red staining)	**Dimensional Properties:** Diameter **Viability:** Mitochondrial and membrane potential, calcium concentration **Other:** RNA-seq	Guanxinning injection [81]	[82] [81]
	Two-cell-type spheroids	CF and CM (Mice) [83] (Human) [84]	TGF-β Pre-culture fibroblasts in stiff plastic before assembling them in spheroids [84] CM:CF ratio	**Cell Markers:** α-actinin, vimentin **ECM Remodeling:** Fibronectin, laminin **Mechanical Properties** **Dimensional Properties:** Spheroid volume **Secreted Molecules and Pathways:** Upregulation of TGFβ in media after stimuli, activation of TGF pathway: pSMAD2, MMP-2, and MMP-9 activities	**Dimensional Properties:** Spheroid volume **ECM Remodeling:** FN, Laminin, TIMP-1 **Activity:** MMP-2 and MMP-9	SB 431542 (TGFB inhibitor) [83]	[84] [83]
	One-cell-type spheroids	CF (Human)	High glucose media + TGF-β	**Cell Markers:** α-SMA **ECM Remodeling:** Collagen type I, MMP-2, and MMP-9 activity	-	-	[85]
	Bioprinted spheroids	CF and CM (Human)	CM:CF ratio (healthy 4:1—scarred 1:4)	**Proliferation and Viability:** Viability **Cell Markers:** α-actinin (sarcomere formation), cTnT, and vimentin (cell distribution and fusion of spheroids) **Functional/Contractile Properties:** Contraction recording, amplitude analysis **Electrophysiological Properties:** Connexin-43 (coupling) **Calcium Handling/Dynamics:** Optical mapping of intracellular Ca^2^⁺ (synchronization), key parameters of the cardiac cell cycle (calcium transient duration, time-to-peak, calcium flux amplitude)	**Proliferation and Viability:** CM and CF proliferation (EdU, Vimentin, and cTnT staining) **Functional/Contractile Properties:** Contraction recording/amplitude analysis **Calcium Handling/Dynamics:** Activation delays, calcium flux amplitude	miR302 b/c induced Hippo inhibition	[86]
**Organoids**		CF, CM and EC (Human)	Hypoxia-induced ischemia + TGF-β [87] Cryoinjury [88]	**Proliferation and Viability:** Sensitivity of ischemic injury and ischemic reperfusion injury (CM apoptosis) **Cell Markers:** Cellular composition and distribution (FACS, Immunostaining, qPCR, and single-cell transcriptomics), integrity of sarcomeric structures **ECM Remodeling:** Collagen deposition, mRNA expression of fibrosis-related genes (ACTA2, POSTN, Vimentin, MMP-2), and collagen-related genes **Dimensional Properties:** Volume **Secreted Molecules:** Release of cTnT, MB, and CKM **Functional/Contractile Properties:** Contraction (arbitrary units) **Calcium Handling/Dynamics:** Calcium signaling and transients **Electrophysiological Properties:** Beating rate, spike amplitude, conduction velocity, field potential duration corrected by frequency (multielectrode arrays)	**Functional/Contractile Properties:** Contraction (arbitrary units) **Calcium Handling/Dynamics:** Calcium signaling and transients **Cell Markers:** α-SMA, VIM, CDH5, PECAM1, TNNT2, MYH7 **ECM Remodeling:** Connective tissue deposition **Secreted Molecules:** Secreted cTnT	Captopril [88]	[88] [87]
**dECM**	Layer-by-layer 3D extrusion printed scaffold with cdECM Bioink, Laponite-XLG nanoclay, and PEG-DA [89]	CF and CM (to assess biocompatibility) (Human)	Stiffness	**Rheological Characterization of Bioink Formulations:** Yield stress, loss factor, tan δ, viscosity **Gelation Kinetics:** Rate and behavior during gelation **Bioink Stability:** Filament width at different timepoints **Cell Viability**	-	-	[89]
	Fibrotic dECM from activated iPSC-CF [90]	CM and CF (to produce ECM) (Human)	TGF-β (to stimulate the CF producing the matrix)	**Cell Markers:** Purity of differentiated iPSCs: Matrix-Producing Cells (CF): α-SMA, fibroblast activation protein (FAP), vinculin; Seeded CM: α-actinin (sarcomere assembly), cTnT **Secreted Molecules:** TGF-β **Characterization of CF-Produced dECM: Morphology:** Collagen type I/type IV, fibronectin, ED-A and ED-B fibronectin isoforms, laminin **Matrisome Components:** Mass spectrometry **Mechanical Properties:** CM behavior/organization: number, proliferation, adhesion (YAP nuclear/cytoplasmic ratio), sarcomere assembly **Functional Properties:** Beating activity, contraction amplitude, contractility traces **Calcium Handling/Dynamics:** Calcium uptake, transient amplitude, and decay time	**CM Behavior/Organization:** Sarcomere assembly	Irreversible inhibitor of LOX, beta-aminopropionitrile (BAPN) (reduces tissue stiffness) → Used on the CF producing the dECM	[90]
	Porcine dECM [85]	CF (Human)	High glucose media + TGF-B + mechanical stimulation (cyclic pressure)	**Proliferation and Viability:** Viability **Cell Markers:** α-SMA **ECM Remodeling:** Collagen type I, MMP-2, and MMP-9 activity	-	-	[85]
**Models on a chip**	Microtissues attached to PDMS rods/micropillars	CF and CM (Human)	CM:CF ratio (healthy 3:1-fibrotic1:3) + TGF-β [91] Laser injury [92]	**Cell Viability and Proliferation:** Cell apoptosis, necrosis, proliferation **Cell Markers:** CM alignment, α-SMA positive cells distribution, vimentin, fibronectin **ECM Remodeling:** Fibrillar collagen deposition, collagen type I/III ratio **Mechanical Properties:** Young’s modulus, passive tension **Secreted Molecules:** BNP secretion **Electrophysiological Properties:** Excitation threshold **Calcium Handling/Dynamics:** Calcium transient amplitudes and synchronization **Functional/Contractile Properties:** Beating pattern, twitch force	**Secreted Molecules:** BNP secretion **Mechanical Properties:** Stiffness, passive tension **Functional/Contractile Properties:** Active force **ECM Remodeling:** Collagen deposition **Other:** mRNA, miR signature	Carvedilol, Losartan [91]	[91] [92]
	Biowire (fibrin-based hydrogel fitted with two polymer (POMaC) wires subjected to electrical conditioning)	CF and CM (Human)	CM:CF ratio (healthy 3:1-fibrotic1:3) + TGF-β1	**Cell Markers:** α-SMA positive cell distribution **ECM Remodeling:** Collagen deposition and alignment (second harmonic generation imaging) **Structural/Mechanical Properties:** Sarcomeric length, Young’s modulus, passive tension **Electrophysiological Properties:** Excitation threshold, action potential profiles, connexin 43 **Functional/Contractile Properties:** Maximum capture rates, force–frequency relationships, post-rest potentiation (PRP) of force **Calcium Handling/Dynamics:** Calcium transient amplitudes and synchronization	**Mechanical Properties:** Passive tension (main screening parameter) **ECM Remodeling:** Collagen imaging and tissue compaction data (validation) **Functional/Contractile Properties:** Active force, ET, and MCR (monitoring)	Furin inhibitors: PCI(p-guanidinomethyl-phenylacetyl-Arg-Val-Arg-4-amidinobenzylamide) Fil (dec-RVKR-cmk) Fill (hexa-D-arginine amide)	[93]
	3D microtissues subjected to mechanical stimulation (uniaxial strain)	CF (Human) [47,94] CM and CF (Rat) [95]	Mechanical (uniaxial strain) and biochemical stimulation (TGF-β)	**Cell Viability and Proliferation:** Proliferation **Cellular Markers:** α-SMA, cTROP **Cellular Populations Distribution** **ECM Remodeling:** Aggrecan, fibronectin, collagen type I, collagen type III, tenascin-c, MMP-2, Nppa **Secreted Molecules:** TGF-β **Electrophysiological Properties:** Excitation threshold **Functional/Contractile Properties:** Maximum capture rates, contraction direction, contraction probability density function **Expression of Proteins Involved in Fibrotic Pathways:** RhoA/ROCK pathway, hippo pathway, WNT pathway, TGFB pathway, CTGF, PAI-1, BNP, NPRA	**Cellular Markers:** α-SMA **ECM Remodeling:** Aggrecan, fibronectin, collagen type I, collagen type III **Others:** CF reprogramming into CM-like cells [47]	Pirfenidone [47] Tranilast [47,94] miRcombo(miR-1,miR-133,miR-208,andmiR-499) [47]	[47] [94] [95]

Single-cell-type 3D models are rare and usually consist of CF (Table 1). Exceptions include models like the one described by Niro et al. [90], which only includes CM. However, even in this example, the CM are cultured in a decellularized ECM (dECM) synthesized by CF, emphasizing the critical role of CF in fibrosis modeling. Most 3D models combine CF with CM, and in some cases EC, adding physiological relevance to the model by incorporating contractility, electrical signaling and intercellular interactions (Table 1).

Importantly, when these cell types are combined, the ratio of CM/CF is of utmost importance and can be adjusted to simulate either healthy or profibrotic conditions. A ratio of 3:1 (CM to CF) is usually used to mimic healthy tissue and 1:3 to mimic fibrotic tissue [76,91,93].

### 4.2. Profibrotic Stimuli

Key soluble signals responsible for fibroblast activation include growth factors such as TGF-β and PDGFs, cytokines like IL-6 and TNF-α, and hormones such as aldosterone and Ang II [30]. Among these, TGF-β stands out as the most critical regulator of myofibroblast activation, which explains its frequent use as a profibrotic inducer in in vitro models (Table 1).

Interestingly, some models utilize Brain Natriuretic Peptide (BNP) as a profibrotic stimulus, despite its well-documented antifibrotic effects [96]. This apparent contradiction can be explained by regional differences in BNP activity because its antifibrotic properties appear to be confined to the ventricle, whereas in the atrium, BNP exhibits a profibrotic effect [97]. As such, studies employing BNP as a profibrotic stimulus have primarily focused on atrial CF, where its actions align with the atrium-specific fibrotic processes.

In addition to biochemical signals, mechanical cues (such as stiffness, topography, or dynamic strain) can also be modulated to maintain CF quiescence [26] or induce myofibroblast activation [79]. A curious example is a 3D model that uses 2D TCP (stiff substrates) to induce CF activation to later assemble them in fibrotic spheroids by using a hanging drop plate [84]. Others directly culture cells in a stiff 3D biomaterial to promote myofibroblast activation. Beyond using direct stimuli such as biochemical or biomechanical cues, many manipulate the ratio of cell types, particularly the CM:CF ratio. An elevated number of CF acts as an indirect profibrotic stimulus, as it mimics CF proliferation, leaving more CF ready to be activated and produce excessive ECM or profibrotic molecules such as TGF-β, while impairing the electrical and contractile behavior of the microtissue.

Alternative approaches, aiming to better mimic pathophysiological conditions, cause microtissue damage through methods like cryoinjury [88], hypoxia-induced ischemia + TGF-β [87], or laser injury [92], bringing them closer to the fibrotic process that occurs after myocardial damage.

## 5. Exploring Cardiac Fibrosis Using 2D In Vitro Models

2D models provide a simple and controlled set-up to evaluate drug effectiveness and toxicity, as well as study fundamental biological processes. However, due to their reductionist nature, they often lack biological complexity and lose important information that influences cell behavior. Thus, 2D models are usually used alongside other approaches. They can serve as an initial test tool, as they are used in high-throughput in vitro platforms [54,55], primarily to identify potential drugs in a cost-effective manner, as further discussed in Section 5.3. Alternatively, they can be used as a confirmatory test after an in vivo experiment, validating molecular and cellular mechanisms of action of a certain drug [70,71]. This is particularly useful when findings from in vivo models remain inconclusive due to the complexity of whole-organism systems, where multiple interacting factors may obscure direct cause-and-effect relationships. Furthermore, they can also serve to understand basic biological processes occurring at the cellular level during fibrotic events [98].

### 5.1. Cell Culture Platforms

2D models typically use standard TCP plates. Since CF respond to mechanical cues and become activated in substrates of higher stiffness [99], the surface of TCP plates can be coated with commercial ECM components such as collagen, gelatin, or gelatin–fibronectin to minimize uncontrolled CF activation during cell maintenance. For high-throughput studies, these platforms are miniaturized (96 or 384-well plates) and often coupled with automated and robotized pipetting, enabling rapid and cost-effective screening of multiple compounds [54,55].

### 5.2. Model Characterization and Readout Parameters

2D models reduce disease complexity to provide a controlled setup for studying specific molecular mechanisms, addressing targeted research questions, and conducting drug screening. Therefore, it is paramount to characterize the model and ensure that it resembles the characteristics of the specific research objective.

Most 2D models focus on the differentiation from CF to myofibroblasts and examine how different drugs influence this process. These assays typically expose CF to a profibrotic stimulus and assess signatures of myofibroblast activation (Table 1), namely cytoskeletal reorganization and formation of stress fibers (α-SMA, F-actin filaments, and phalloidin) [59]. Additionally, secretion of ECM structural proteins such as collagen type I (procollagen or mature trimers), collagen type III, and fibronectin are also commonly assessed. The expression, localization, or activity of these markers is typically analyzed by immunofluorescence, qPCR, ELISA, Western blotting, proteomic analysis, gelatin zymography (for MMPs), or the combination of these techniques. However, standard cell culture conditions fail to recapitulate the dense molecular environment present in vivo. To address this, some models [55] have adopted the main principle from the “Scar in a Jar” classical model [100], and used different macromolecules, such as dextran sulfate (DxS) or neutral mixed Ficoll, to simulate crowding, thereby accelerating collagen deposition and maturation. Furthermore, confirming that collagen deposition occurs specifically in the extracellular space is essential and can be achieved using immunofluorescence to label extracellular glycosaminoglycans linked to collagen or through scanning electron microscopy (SEM) to visualize secreted collagen fibers [55]. Besides structural ECM proteins, other matrisome components such as POSTN [55] and MMP-2/9 [67,72], have been also investigated.

Beyond myofibroblast differentiation and ECM analysis, some models also study the proliferative or migratory capacity of CF [71]. Models with a particular interest in specific molecular targets or pathways also evaluate the expression of certain receptors such as TGF-βR or the antifibrotic factor hepatocyte growth factor (HGF) receptor (c-met) [67], as well as levels of intracellular reactive oxygen species (ROS) or inflammatory mediators when testing anti-inflammatory drugs [69]. For models including CM, an assessment of contraction–relaxation is also commonly performed [67].

Altogether, the goal of this characterization is to determine which culture conditions (different stimuli, dose, or timepoint) yield a better representation of the fibrotic environment when compared to non-stimulated controls (healthy control). Once the model is established and confirmed to display distinct characteristics compared to healthy controls, the next step involves testing therapeutic candidates. Drug testing relies on readout parameters that largely overlap with those used during model characterization (Table 1), including the expression of matrix remodeling genes (collagens, assembly proteins, and proteases), mesenchymal markers (α-SMA, fibronectin, vimentin), as well as fibroblast proliferation and migration rates. In cases where a drug is intended to target a specific pathway, additional readout parameters may be introduced to reflect the targeted mechanism, such as ROS reduction for oxidative stress inhibitors or Wnt/β-catenin signaling markers for pathway-specific treatments. Transcriptomic analyses may also be employed to assess the drug’s impact on the broader expression of fibrosis-associated genes. Positive controls, such as well-characterized antifibrotic drugs like pirfenidone, are critical to validate the model by demonstrating that its fibrotic traits can be prevented or reversed [54].

### 5.3. High-Throughput Screening in 2D Models

One of the main advantages of 2D models is their easy high-throughput scalability. High-throughput screening (HTS) is the method of testing various substances in a different assay. Typically, these screenings can identify new targets or drugs in more than 10,000 assays a day [101]. The goal of HTS is to identify a small set of compounds with the desired biological activity (true positives) from a vast collection of “null” compounds, which exhibit little or no biological activity (true negatives). Thus, to be suitable for HTS, assays must be both robust and economic. The robustness of HTS can be evaluated with metrics such as the Z’ factor [102,103]. Positive and negative control values should be smaller than a threshold defined as the mean of all values plus three times the standard deviation. An ideal HTS assay would have a Z’ factor of 1, but a good assay is defined by a Z’ factor larger than 0.5.

In a common HTS drug assay, a cell-based screening is used to test a large library of compounds. Following the primary screening, potential hit compounds are validated through secondary testing, which includes determining their half-maximal inhibitory concentration (IC50) to assess potency [104]. There are several detection methods for HTS, including enzymatic activity, luminescence, and fluorescence [105,106]. High-throughput imaging (HTI) is a commonly used and informative detection method. A typical HTI pipeline requires automated handling of reagents for assays and staining, and automatic image acquisition and analysis [107]. Typically, features from fluorescence images collected by widefield or confocal microscope in multi-well plates are extracted and quantified. Besides screening, HTI can be used for profiling and deep imaging [107]. HTI profiling leverages the ability to generate large, multiparametric datasets of cellular features through automated image analysis and statistical learning techniques, enabling the classification and clustering of treatments based on the similarities in the phenotypes they produce. In deep imaging approaches, the vast amount of single-cell data generated by HTI are used to thoroughly investigate the effects of a limited set of experimental perturbations—usually ranging from 10 to 100—on much larger cellular populations (up to 5 × 10⁵) compared to typical HTI methods. This enables the identification and study of rare, yet significant, biological events.

A few recent HTI platforms using 2D models of CF are emerging. One study used colorimetric detection of bromodeoxyuridine (BrdU) to measure CF proliferation following exposure to 480 natural compounds [75]. In another work, a library of 5000 drugs was screened using HTI in iPSC-derived CF engineered to express fluorescent α-SMA, enabling the detection of activation and toxicity together with Hoechst staining [54]. However, these screens primarily correlate overall α-SMA expression with staining intensity, potentially overlooking the organization of stress fibers. The arrangement of α-SMA into stress fibers is crucial, as it underpins many phenotypic behaviors associated with myofibroblast activation. Consequently, stress fiber organization represents a critical aspect of accurately classifying myofibroblast phenotypes [108]. This is why some studies relied on machine-learning algorithms to train models to detect morphological features of activated fibroblasts. Nelson et al. validated a previous in silico study in CF and classified cells into activated or non-activated according to the intensity and texture of α-SMA [73]. Palano et al. developed a drug screening platform validated with known anti-fibrotic compounds in CF and classified cells based on the expression of both α-SMA and telocollagen 1α1 [55]. Ultimately, integrating rapid detection of multiparametric readouts with unbiased classification presents a promising approach for utilizing high-throughput screening in cardiac fibrosis models.

## 6. 3D Engineered In Vitro Models of Cardiac Fibrosis

3D models aim to go a step closer to the biochemical, biomechanical, electrophysiological, and contractile properties of the human myocardium. By adding an extra dimension, 3D models elevate the biomimetism of in vitro models and translatability to human physiology.

For this reason, most 3D models incorporate CM into the model, allowing the assessment of additional parameters such as abnormal cell coupling and contractibility, alongside myofibroblast activation and ECM remodeling, as reviewed in Section 5.2. Consequently, their action potential profiles are carefully studied, including excitation threshold, conduction velocity, maximum capture rates, force–frequency relationship, post-rest potentiation of force, calcium transient amplitudes and synchronization, and connexin 43 [87,91,93]. These factors are important because they can be altered by ECM deposition or altered gap junction coupling, both expected to be dysregulated in fibrotic models. In terms of contractibility, some advanced models have incorporated sensors for dynamic, accurate, and real-time readouts [76,93]. Additionally, models such as the one developed by Niro et al. demonstrate how fibrotic ECM also influences CM morphology and sarcomere assembly [90].

This increased biological relevance comes with added complexity, requiring further characterization. Most of these models provide a substrate for cell growth, with biomaterials typically characterized in terms of mechanical properties (e.g., Young and compressive Modulus) [93], mass swelling ratio [26], protein-grafting efficiency (in the case of coated scaffolds), water-contact angle, pore size, nanofiber orientation [79], and degradation rate [77]. Ultimately, cell–biomaterial interactions are evaluated in terms of biocompatibility, distribution, migration, or degradation of the matrix (Table 1).

### 6.1. Hydrogels and dECM Scaffolds

The simplest engineered model involves culturing cells in biomaterials with varying stiffness to replicate the mechanical properties of a healthy or injured matrix and control myofibroblast activation. For instance, Zhao et al. produced a hydrogel with regions of 10% and 20% polyethylene glycol diacrylate (PEGDA) to emulate the different stiffnesses found in healthy and infarcted tissues, respectively. By evaluating cellular markers, collagen deposition, and cell migration, this model allowed the assessment of fibroblast behavior in response to different substrates, demonstrating how stiffness influences myofibroblast activation and behavior [60]. Another approach consists of encapsulating CF and CM in gelatin methacryloyl (GelMa) hydrogels with adjustable stiffnesses, designed to replicate the properties of native heart tissue, thereby preventing spontaneous myofibroblast activation and maintaining fibroblast quiescence [26,77]. By subsequently adding TGF-β, researchers can induce myofibroblast activation, isolating the effects of biochemical signaling without the confounding factor of substrate stiffness.

ECM decellularization, which combines physical, chemical, and enzymatic processes to remove cells while preserving ECM composition and functionality [109], provides a more complex tissue culture platform than hydrogels, including both the biomechanical and biochemical cues found in the native/fibrotic tissue. The classical approach consists of decellularizing animal ECM, usually porcine, and repopulating it with CF to assess their response to profibrotic factors in a native-like environment [85]. However, dECM-based biomaterials use is hampered by the lack of donors, interspecies differences, and batch-to-batch variability. To address this issue, creative solutions like the one proposed by Niro et al. have emerged, in which the authors used substrates of different stiffness to generate hiPSC-CFs preparations with different activation levels and produce fibrotic ECM [90]. Subsequently, the synthetized ECM was decellularized and repopulated with hiPSC-CM to assess CM behavior in fibrotic human dECM. Furthermore, dECM analysis revealed that the produced matrix contained 352 matrisome components, including structural elements, basal lamina elements, growth factors, and proteins involved in ECM remodeling.

### 6.2. Spheroids and Organoids

Cell–cell and cell–ECM interactions are essential in any biomimetic system, influencing processes such as coordinated gene expression, cell survival, differentiation, and apoptosis. For this reason, 3D-cellular aggregates like spheroids and organoids can be used as models of cardiac fibrosis (Table 1), although it is important to highlight that they differ in origin, complexity, and functionality.

Spheroids are 3D cell aggregates formed by allowing or forcing cells to self-aggregate. They usually include CF and CM, as well as EC to account for the vascularization of the model [82]. By mimicking cell–cell and cell–matrix interactions, spheroids enable cellular proliferation, differentiation, apoptosis, and ECM remodeling. Furthermore, the 3D nature of the model allows us to evaluate aspects such as drug, nutrient, and oxygen gradients, and it can be designed to replicate organotypic and pathological cell densities.

However, spheroids lack the tissue-specific structure and cellular organization seen in natural development. Instead, organoids are 3D structures grown from stem cells that, guided by diverse signaling cues, differentiate and self-organize to mimic the architecture and functionality of specific organs or tissues. This closer resemblance to in vivo systems makes them more reliable. However, it is important to highlight that at initial stages, an organoid will always mimic the healthy heart, because organoids recapitulate developmental processes unless the organoid is derived from iPSCs from an individual with a cardiac congenital disorder [110]. Thus, these healthy organoids need to be instructed to recapitulate fibrotic events. For example, Yang J et al. used cryoinjury to mimic myocardial injury [88], while other approaches have induced ischemia and ischemia–reperfusion (IR) injury by treating the organoids with cobalt chloride (CoCl_2_) and subsequently exposing the organoids to TGF-β [87].

### 6.3. Bioprinted 3D Models

Bioprinting allows precise placement of cells and biomaterials aiming at recreating tissue structures with a defined architecture. Moreover, bioinks can be tuned to modulate the mechanical and biochemical properties of constructs, constituting a relevant tool for developing novel fibrosis models. For example, Basara et al. bioprinted a model with three distinct regions mimicking remote, border, and scar regions of a post-MI aged myocardium [111]. To do so, three unique bioinks were designed by varying the proportions of cardiac cells (CM, CF, EC, and myofibroblasts) and incorporating distinct hydrogel compositions for each region, including GelMA, MeHA (methacrylate hyaluronic acid), collagen, and photoinitiators. Another smart approach is the model developed by Daly et al., in which CM and CF ratios were spatially controlled to bioprint both ‘healthy’ and ‘fibrotic’ spheroids. These spheroids were then fused using a self-healing hydrogel to create heterogeneous microtissues that mimicked a scarred heart. Through immunofluorescence analysis and assessment of microtissue contraction and calcium signaling, incorporation of one or more fibrotic spheroids in the construct significantly impaired the functionality of the construct by reducing contractile output and disrupting electrical synchronization [86].

During fibrosis, the increase in ECM deposition leads to progressive architectural disruption, which causes CM hypertrophy and anisotropy loss. Building on this phenomenon, bioprinting techniques like electrospinning have been used to create scaffolds that mimic the disorganized structure and increased stiffness of fibrotic tissue. For example, different polycaprolactone (PCL)-based scaffolds with randomly oriented fibers, coated with ECM proteins (e.g., fibronectin, laminin, collagen type I and type III, tenascin C) have been fabricated [79,80]. Remarkably, these models have shown that increased stiffness and topographical cues (randomly oriented nanostructures) can activate hCF [79].

dECM is also an interesting material for developing bioinks, as it may preserve growth factors, ECM proteins, and glycosaminoglycans (GAGs) specific to the source tissue [89]. Some models have developed bio(material)inks composed of partially digested dECM from human [112] or porcine sources [89], which are usually used in combination with other biomaterials such as Laponite-XLG nanoclay and poly(ethylene glycol)-diacrylate (PEG-DA), which improve the rheological properties necessary for printing [89]. Furthermore, the mechanical properties of dECM-based bioinks can be modified to mimic pathologic conditions like fibrosis. However, incorporating non-degradable materials into the bioink formulation may hinder cells from degrading and remodeling the matrix, which is a major limitation of these approaches.

### 6.4. Specialized Models on a Chip

Simple 2D models mainly focus on CF, their activation, proliferation, and excessive ECM deposition. In the heart, this excessive ECM can create conduction barriers that disrupt electrical coupling, while the overabundance of myofibroblasts can form heterocellular gap junctions, leading to abnormal impulse propagation and altered contractility [113]. Specialized 3D platforms have now been engineered to better capture and or recreate these features in fibrotic models (Table 1).

Although 3D models including CM and CF often exhibit contraction, this is generally not considered a fibrosis readout, given that sophisticated equipment and complex analyses are required to measure cardiac force [78]. To address this limitation, specialized platforms have incorporated biosensors or micropillars/rods whose deflection gives an easy quantification of contractility. An example is the microtissue developed by Shang et al., which consists of different ratios of CF and CM seeded on a structural color hydrogel with biomimetic topography (microgrooves). The structural color of hydrogel changes color due to the interaction of its nanoscale structure and light. When cells contract, they physically pull the hydrogel, slightly compressing or stretching its nanoscale structure. These changes modify the way the hydrogel reflects light, shifting the observed color. Then, the contractile behavior of the healthy, fibrotic, and treated (with potential drugs) microtissue can be easily assessed by observing the color changes in the hydrogel substrate during cell activity [76].

Another example is the Biowire II platform, a model of interstitial and focal fibrosis [93]. This model evolved from the Biowire platform, originally designed to mature hiPSC-CM by combining 3D cell culture with electrical stimulation. The original platform embedded cells in a collagen gel around a template suture in a microfabricated well and subjected them to electrical field stimulation with progressive frequency increase [114]. In the Biowire II, a microtissue composed of a fibrin hydrogel containing both CM and CF is suspended between two elastic wires made of POMaC (poly(octamethylene maleate (anhydride) citrate)). When the microtissue contracts, the wires deflect, enabling real-time measurement of active force, passive tension, calcium signaling, and electrical properties. For accurate modeling, it is crucial that microtissues containing CM show proper maturation and synchronous contraction patterns [115]. The Biowire II [93] platform facilitates this by using electrical conditioning to support tissue maturation, which allows for drug testing at different time points, including both the early and later stages of scar formation. Notably, research using this model has shown that certain drugs can be more effective when administered in the early stages of fibrosis, highlighting the importance of timely intervention to maximize therapeutic outcomes. Furthermore, advancements in this system have created heteropolar Biowires, that combine scar-like and healthy tissue regions within the same structure or heteropolar cardiac tissues containing distinct atrial and ventricular ends [116].

Some other models have also introduced an additional layer of complexity by incorporating mechanical stimulation. Dynamic mechanical tension has been reported to induce the phenotypic transition from fibroblasts to myofibroblasts. Based on this principle, various models have employed mechanical stimulation in combination with biochemical stimulation, such as TGF-β, to trigger fibrosis [47,94]. In contrast, other models have shown how CM can attenuate the profibrotic effects of cyclic stretch and TGF-β on CF [95]. One notable platform is the uScar, which provides a physiological uniaxial strain of 10% to fibroblast-laden fibrin gels [47]. This mechanical stimulation has been shown to increase the expression of proteins implicated in the RhoA/ROCK pathway while also triggering myofibroblast activation (α-SMA) and ECM deposition. In the same work, uniaxial strain was also found to negatively impact the direct reprogramming of CF into induced cardiomyocytes using miRNAs, a result that contrasts the success of reprogramming in static models [117]. This work supports that mechanical stimulation is an important parameter to consider when assessing the efficacy of drugs/therapies in 3D bioengineered cultures.

## 7. Bridging the Gap Between High Throughput and Biological Relevance

An ideal model will balance biological relevance, reproducibility, and high throughput capabilities. However, this balance can be tilted to one side or the other depending on the scientific question. As mentioned, the ideal model should include relevant cell types as well as biochemical and biomechanical cues, represent the hallmarks of fibrosis (fibroblast activation, excessive ECM deposition, and impaired electrical coupling and contractibility), and have clear, measurable, and objective readouts. However, greater complexity often entails lower throughput (Figure 3).

On one hand, 2D models are a simplified version of the complexity comprised in fibrotic events; they often provide reproducibility, cost-effectiveness, and high-throughput capabilities. On the other hand, 3D models often exhibit greater biological relevance, but this comes with complexity, confounding effects, and less throughput. The challenge is to combine the best of both worlds by designing a biologically relevant system with high-throughput capabilities. Achieving this will imply the combination of bioengineering, high-level automation, and artificial intelligence (AI) methods.

Out of the 3D models described herein, organoids and spheroids are the easiest to translate into high-throughput platforms. Spheroid and organoid assembly already requires the fabrication of multiwell microarrays of hydrogels or polymers. These platforms are more amenable to HTI by confocal microscopy [118]. The use of light-sheet fluorescence microscopy has revolutionized 3D imaging, enabling minimization of bleaching and light penetration issues related to conventional confocal imaging, which can be adapted in the future for HTS. To date, no works have been developed using spheroids or organoids to screen cardiac fibrosis in high-throughput assays. Interestingly, despite being more complex, some works have applied HTS to screen contractility in engineered heart tissues, but not cardiac fibrosis.

With the recent advancements in AI, the focus is now shifting to developing bioengineered platforms that support automation and high throughput, and technologies like sensors and microfluidics are already paving the way. A clear example is the model proposed by Y. Shang and colleagues, in which a structural hydrogel capable of changing its color serves as a visual readout of contractibility [76]. This biomimetic fibrotic microtissue was integrated into a microfluidic system with a concentration gradient generation (CGG) network, thereby increasing the throughput capabilities of the model. Another example is the use of 3D-printed microphysiological devices that enable continuous electronic readout of the contractile stress of multiple laminar cardiac micro-tissues [119]. Each chip in this system contains eight wells, with each well incorporating a multilayer cantilever, a strain sensor, and a tissue-guiding layer. This set up facilitates the self-assembly of physio-mimetic laminar tissues of CF and CM in a miniaturized platform, allowing for both biological relevance and high-throughput automated testing on a single platform. Although this model was not designed for fibrosis modeling, by introducing the right profibrotic stimuli, it could be easily adapted.

Altogether, the selection of the most appropriate cardiac fibrosis model requires a thoughtful consideration of the scientific question and objectives. If the goal is to investigate a specific mechanism or conduct initial screenings with a large number of compounds, 2D models—which reduce the amount of confounding effects and are technically less challenging and cost effective—are probably the best option. On the other hand, when the goal is to understand how certain cellular types behave in an in vivo-like setting or evaluate the potential of a drug in a model that closely resembles the human body, 3D models are the way to go. If we seek to combine biological-relevance with high-throughput capacities, we can leverage bioengineering to develop models that combine the best of both worlds.

## 8. Tested Molecules in In Vitro Systems

Some drugs like pirfenidone (TGF-β inhibitor) or nintedanib (tyrosine kinase inhibitor) are well-known inhibitors of myofibroblasts activation. For this reason, they are commonly used as model antifibrotic molecules to test the sensitivity of the platform. Other tested drugs have been summarized in Table 1, and include tranilast (TGF-β inhibitor) [79], losartan (Ang-II receptor blocker), saracatinib (kinase inhibitor), relaxin-2 (peptide hormone) [76], ONO-1301 (prostacyclin agonist), camostat mesilate (serine protease inhibitor) [67], or SB431542 (ALK 4/5/7 inhibition) [55]. Some models have also tested furin inhibitors [93] or ROCK inhibitors [60], as well as strategies that are used to treat other cardiac conditions like coronary artery disease (Guanxinning injection (GXNI)) [81]. Besides GXNI, other natural compounds such as ellagic acid and punicic acid [69], quercetin dihydrate [71], bufalin and lycorine [75], or curcumin [72] have also demonstrated their antifibrotic potential in in vitro platforms. Additionally, due to the simplicity and high-throughput capabilities of 2D models, they have been used in screening assays to test compound libraries [54,75].

In addition to small molecules and natural compounds, miRNAs and cellular components have also been evaluated. For example, delivery of miR302 b/c (which interferes with the Hippo pathway) improved the functional properties of scarred spheroids [86]. Others have aimed to deliver various miRNAs (miRcombo: miR-1,miR-133,miR-208,andmiR-499) to fibrotic microtissues to assess whether the combo was capable of inducing direct reprogramming of fibroblasts into CM-like cells in this fibrotic environment [47]. The antifibrotic effect of specific secretomes has also been studied on these platforms [120]. For example, a GelMa hydrogel containing human fetal CF served as a platform to test the paracrine inhibitory effects of cardiac progenitor cells on both CF activation and collagen synthesis [26].

## 9. What Can We Learn from Other Organs?

Aside from myocardial conditions, fibrosis is a common mechanism present is many diseases affecting other organs such as the lungs, liver, kidneys, skin, and intestine, where it also leads to organ dysfunction.

### 9.1. Conserved Mechanisms and Inspirational Molecular Targets

Each organ has a different physiology and a different cellular response to fibrotic triggers. In vitro fibrosis models are adjusted to the organ of interest regarding cell types, platforms, stimuli, and readouts. However, fibrosis shares common mechanisms and molecular pathways, enabling transversal research across multiple organs. A clear example is how pirfenidone, initially investigated and already approved for idiopathic pulmonary fibrosis (IPF), is now one of the most promising antifibrotics for cardiac applications. Similarly, other drugs like Nintedanib/tyrosine kinase inhibitors [121], statins [122], or pyridoxamine (kidney) [123], have also been explored in other organs and subsequently shown therapeutic benefits in cardiac fibrosis [124,125]. Interestingly, molecular targets identified in other systems, like lysyl oxidase-like 2 [126], lysophosphatidic acid receptors [127], 5-hydroxytryptamine receptors [128], and PPARγ pathway [129], are now being studied in the context of the heart [125].

Although most cardiac antifibrotic research has been mostly focused on the renin–angiotensin–aldosterone system or TGF-β pathways, insights from fibrosis research in other organs could be repurposed for the heart. For instance, in the liver, caspase inhibitors have been demonstrated to induce HSC apoptosis, reduce TGF-β levels [130], and subsequently mitigate fibrosis [131]. Given that caspases are apoptosis mediators common to most organs, their inhibition could yield similar results in the heart. However, this needs to be confirmed, for instance by capitalizing on in vitro models to validate whether caspase inhibitors can selectively induce cardiac myofibroblast apoptosis without affecting non-activated CF or CM.

Another promising approach involves inhibiting C-C chemokine receptor type 2/5 (CCR2/5), which mediates the profibrotic effect of monocytes and macrophages in various organs [132,133], including the heart, where CCR2 plays a profibrotic role [134]. While CCR2 inhibitors have been researched in liver, renal, and chronic colitis-associated fibrosis [135], their potential in cardiac fibrosis remains unexplored. This gap likely arises from a common limitation in cardiac fibrosis models; that is, the lack of representation of the immune compartment, which is a main orchestrator of the fibrotic response, regulating the balance between profibrotic and antifibrotic signals [35].

### 9.2. Platforms and Culture Conditions

Numerous IPF in vitro models incorporate immune cells [136]. For instance, 2D models expose immune cells to conditioned media from lung fibroblasts and vice versa, while other systems use transwell co-cultures [137]. These simple yet valuable studies have demonstrated how, for example, antifibrotics can decrease B-cell-mediated fibroblast migration and activation [138]. More advanced IPF 3D models have co-cultured fibroblasts and immune cells on hydrogels in dECM [139] or have employed transwell systems where cells are cultured on both sides of the membrane, deviating from the classical configuration of separate compartments in transwell systems [140]. These co-culture systems have determined that the co-culture of mast cells and fibroblasts leads to greater fibronectin and collagen type I deposition, mainly mediated by an increased release of the enzyme tryptase by mast cells [141]. Another example is liver fibrosis models, in which the inclusion of Kupffer cells in bioprinted liver constructs was shown to delay hepatocyte injury under TGF-β stimulation [142]. These findings highlight the importance of integrating the immune system in cardiac fibrosis models, which will require optimization of cell culture conditions required to maintain viable and functional cardiac and immune cells.

In IPF, the alveoli are the primary site of injury, making them critical for disease modeling. Many pulmonary fibrosis models replicate this air–liquid interface, such as transwell systems where cells on one side of the membrane are exposed to air, while those on the opposite side receive appropriate nutrition and dynamic stimulation [143]. These air–liquid interface cultures have been instrumental in studying the crosstalk between epithelial cells and fibroblasts, as well as epithelial-to-mesenchymal transition [144]. Although the heart lacks a comparable air–liquid interface, a similar approach could provide insights into the interactions between its myocardial and endothelial layers, along with the impact of blood flow dynamics. For example, a scaffold resembling myocardial tissue could be seeded with endocardial cells on one side and integrated into a system with pulsatile flow. The setup could replicate blood flow across the endocardial layer, simulating shear stress and conditions such as cardiac pressure overload, enabling the involvement of EndMT transdifferentiation in fibrotic events. This transition, specifically when triggered by mechanical stimuli, remains poorly understood [30] and, to our knowledge, has not yet been studied using in vitro models [145].

The role of flow in influencing cellular behavior has also been studied in liver fibrosis models. Liver-on-a-chip systems simulating hepatic sinusoidal flow dynamics have demonstrated that mechanical stimuli induced by medium flow significantly impact cellular differentiation [146], pinpointing the importance of evaluating similar outcomes in cardiac fibrosis models. Moreover, representing the structural organization of blood vessels in the heart, where the blood first penetrates a layer of EC and then enters the cardiac parenchyma, would be ideal for understanding how excessive ECM deposition affects drug penetration. For example, liver fibrosis models have used gelatin-based bioinks as a sacrificial material to sequentially deliver different cell types, fabricating a microscale multilayer structure [147]. Nevertheless, the cardiac field is making significant progress, with the construction of 3D-printed thick, vascularized, and perfusable cardiac patches already being a reality [148]. The goal now should be to mimic fibrosis in these types of systems to understand how it affects drug penetration and investigate delivery strategies for enhanced outcomes.

In addition to providing tissue support, the ECM is an important reservoir of signaling molecules in homeostasis and disease across organ systems [149,150]. While cardiac fibrosis models working with dECM-based materials could be a step closer to this consideration [90], the role of matrikines is yet to be explored in this context. In the lung, the release of proline–glycine–proline (PGP) from collagen cleavage has been shown to promote neutrophil chemotaxis. Thus, strategies to increase the degradation of PGP (or acetylated PGP, a modification induced by cigarette smoke) have been investigated in order to limit the inflammatory response [151]. While the heart is not exposed to cigarette smoke, various triggers could lead to the release of profibrotic matrikines, an approach that has been explored in only a few studies [152].

In organs like the liver, lung, or kidney, precision-cut slices have been widely used [153,154]. These ultrathin preparations preserve the structural, biochemical, and electrophysiological properties of native tissue and allow the study of heterocellular interactions and cell–ECM interactions. While cardiac slices have been explored to some extent [155,156,157], there is very little research on cardiac fibrosis using this model. One of the few models using human and canine myocardial slices showed how they are a great platform to culture CF without inducing α-SMA expression and that their proliferation can be modulated with the application of mechanical load, resembling physiological behavior [158]. Another study tested how mechanical load-induced fibrotic phenotypes and how a TGF-βR blocker could prevent overload-induced fibrosis and inflammation [159]. Additionally, there is increasing interest in methods like electromechanical stimulation to extend slice viability [160]. However, precision-cut slices face some challenges [161], and we can learn from how others have solved them. For instance, the cutting of liver slices represents an injury that triggers HSC activation, limiting their application to test-antifibrotic compounds. However, their culture has been optimized by supplementation with valproic acid sodium salt, a histone deacetylase class I inhibitor, which reduced the cut-induced fibrosis while preserving its responsiveness to TGF-β [162], a strategy that could be adapted by myocardial slides.

Altogether, cardiac fibrosis models can learn from platforms addressed for other fibrotic diseases. However, given that cardiac fibrosis is a chronic condition that develops and progresses over extended periods, it will be essential to work towards the longevity of these platforms. For example, liver-on-a-chip models have already recognized the importance of including perfusion systems to optimize nutrient delivery and waste management [163], and heart-on-chip platforms should also prioritize this aspect. Developing such long-lasting models will enable longitudinal studies and a better understanding of the disease pathophysiology, as well as enable the identification of intervention opportunities.

### 9.3. Sensing Systems

Some cardiac fibrosis models integrate sensors to assess the contractibility of the tissue. However, measuring metabolites or secreted molecules often involves the collection of conditioned media and subsequent biochemical analysis to detect the secretion of molecules like TGF-β or BNP. Such an approach has two limitations: these measurements provide data from a specific moment when the media is collected, missing dynamic secretion patterns. Moreover, the miniaturized dimension of on-chip models often results in low concentrations of secreted molecules. However, solutions from other research areas could be adapted for cardiac applications. Son et al. [164] developed a microsystem composed of a microfluidic device and a fluorescent microbead-based assay to detect HGF and TGF-β secreted by primary hepatocytes, which could be adapted to detect molecules secreted from cardiac constructs, such as BNP. Alternatively, liver fibrosis models have also used electrochemical biosensors incorporated in microfluidics to monitor TGF-β production by a co-culture of hepatocytes with stellate cells exposed to alcohol. This system contained miniature electrodes functionalized with TGF-β specific aptamer molecules and enabled the determination of how TGF-β was originally secreted by hepatocytes, which then stimulated the activation of stellate that, in turn, began producing TGF-β of their own [165].

## 10. Conclusions and Future Directions

With CVD still on the rise and their tight relation with fibrosis, new effective treatments are a pressing need. Despite significant advances in understanding the pathophysiology of cardiac fibrosis, effective treatments remain scarce. While animal models offer a good representation of disease processes, they are labor-intensive, resource-demanding, and present interspecies differences. Moreover, the complexity of whole organism systems introduces numerous confounding factors that complicate the establishment of specific correlations. Thus, in vitro models have emerged as simplified, cost-effective, and scalable alternatives that also contribute to reducing animal experimentation in preclinical research by adhering to the 3R principle: Replacement, Reduction, and Refinement.

In vitro cardiac fibrosis models can have three primary purposes: (1) to understand the pathophysiology of fibrosis, (2) to serve as an initial testing platform for drug development and preclinical testing, and (3) to provide mechanistic explanations for drug effects observed in vivo. With these purposes, early 2D models were established to provide a controlled and simplified environment to address specific molecular questions. With the advancements in bioengineering, 2D models have evolved into high-throughput screening platforms, and alternative 3D models have been developed, ranging from hydrogels to beating hearts on a chip.

Despite these advances, significant gaps remain in cardiac fibrosis modeling, particularly in the area of personalized medicine. While patient-derived hCF are valuable for replicating in vitro the disease-associated fibrotic phenotype and studying patient-specific alterations, their use remains limited due to high donor-to-donor variability and restricted access to surgical samples. Some studies have successfully circumvented the need for surgical specimens by differentiating hCF from hiPSCs obtained from patients with specific genetic conditions [64]. A notable example is the study by Soussi et al., where CF derived from hiPSCs of patients with mutations related to myocardial conditions were used to explore how these mutations impact CF behavior and contribute to disease progression [64].

A potential strategy for achieving personalized medicine involves isolating immune cells, such as monocytes, from patient blood samples. These cells are easily obtainable and can be incorporated into in vitro models alongside laboratory-grown CF and CM. This approach could allow for the assessment of how patient-specific immune cells interact with fibrotic microtissues/cells and for testing of antifibrotic drugs, such as those aiming to inhibit macrophage-to-myofibroblast transitions [166]. Another key area of improvement in fibrosis modeling is the standardization of readouts. Current approaches to measuring myofibroblast activation differ significantly between labs, with some measuring α-SMA intensity and others focusing on fiber formation. Standardizing these readouts and implementing artificial intelligence for high-throughput screenings could streamline and improve data analysis.

In addition to modeling fibrosis and drug response, these in vitro systems hold promise for discovering new biomarkers. Biomarkers that increase in fibrotic conditions could be used to monitor disease progression and treatment response, something that has already been explored using in vitro models for some myocardial conditions [167].

In conclusion, cardiac fibrosis models are essential for addressing the burden of CVD. By balancing biological relevance, reproducibility, and scalability, different platforms can be tailored to address specific research objectives, advance the understanding of the disease, accelerate drug development, and precision medicine.
